# Encephalitis, Ontario, Canada, 2002–2013

**DOI:** 10.3201/eid2203.151545

**Published:** 2016-03

**Authors:** Alyssa S. Parpia, Ye Li, Cynthia Chen, Badal Dhar, Natasha S. Crowcroft

**Affiliations:** Public Health Ontario, Toronto, Ontario, Canada (A.S. Parpia, Y. Li, C. Chen, B. Dhar, N.S. Crowcroft);; University of Toronto, Toronto (A.S. Parpia, Y. Li, N.S. Crowcroft)

**Keywords:** encephalitis, incidence, epidemiology, etiology, Ontario, Canada, England, viruses, bacteria, fungi, parasites

## Abstract

Encephalitis, a brain inflammation leading to severe illness and often death, is caused by >100 pathogens. To assess the incidence and trends of encephalitis in Ontario, Canada, we obtained data on 6,463 Ontario encephalitis hospitalizations from the hospital Discharge Abstract Database for April 2002–December 2013 and analyzed these data using multiple negative binomial regression. The estimated crude incidence of all-cause encephalitis in Ontario was ≈4.3 cases/100,000 persons/year. Incidence rates for infants <1 year of age and adults >65 years were 3.9 and 3.0 times that of adults 20–44 years of age, respectively. Incidence peaks during August–September in 2002 and 2012 resulted primarily from encephalitis of unknown cause and viral encephalitis. Encephalitis occurred more frequently in older age groups and less frequently in women in Ontario when compared to England, but despite differences in population, vector-borne diseases, climate, and geography, the epidemiology was overall remarkably similar in the two regions.

## Abstract 2

The epidemiology of encephalitis in Ontario is remarkably similar to that in England.

Encephalitis is a brain inflammation that over the long term can reduce neurologic health and cause disability and even death (*1*,*2*). More than 100 infectious, post-infectious, and immune-mediated conditions can cause encephalitis, which occurs most often in infants and in adults >65 years of age (*3*–*5*). Studies worldwide indicate that cause is unknown for 37%–85% of encephalitis cases and that recorded causes differ by region and implementation of systematized diagnostic algorithms (*3*,*5*–*9*).

Vaccination has reduced the incidence of encephalitis caused by measles, mumps, rubella, and varicella. However, efforts to prevent and reduce infectious and immune-mediated causes of encephalitis must be maintained because the number of possible causes is increasing (*7*). Climate change and increased mobility of humans have contributed to the spread of infectious diseases to newly supportive environments to which such infections are not endemic, ultimately changing the regions in which vectors can transmit various infectious forms of encephalitis (*10*,*11*). Additionally, the increased survival and life expectancy of persons with immunocompromising conditions contribute to the increased incidence of encephalitis. Several studies have identified herpes simplex virus as responsible for the greatest proportion of encephalitis-associated hospitalizations (*3*,*5*,*6*,*8*,*12*), followed by varicella zoster virus (*6*–*8*), or in some studies, *Mycobacterium tuberculosis* (*12*) or *Toxoplasma* meningoencephalitis (*6*).

During 1994–2008, the estimated annual incidence of encephalitis in Ontario, Canada, was ≈4.6 (95% CI 4.5–4.7) cases per 100,000 persons, according to codes recorded based on the International Classification of Diseases (ICD), Ninth and Tenth Revisions (*4*). Encephalitis is a reportable disease according to Ontario Public Health Standards, as are many diseases that can cause encephalitis, such as West Nile virus illness, rabies, and measles (*13*,*14*). However, little is known about the various causes of encephalitis in particular and their category-specific incidence rates and proportions in Ontario. Given the severity of encephalitis, hospitalization data have been found to be reliable for identifying encephalitis incidence, unlike notification data, which yield underestimates due to underchildren-reporting, despite the status of encephalitis as a reportable disease (*4*,*15*). In England, studies have helped identify gaps in understanding and have shown that length of hospital stay varies among categories of encephalitis cause (*7*). England is similar to Ontario in terms of socioeconomic makeup, yet has a starkly different geography. Both have publicly funded healthcare and comparable data available for analysis. Thus, comparison of the incidence of encephalitis in these 2 regions might be telling of region-specific causes. The extent to which hospitalization duration and other measures of illness burden vary among encephalitis causes in Ontario is unknown.

Our objective was to estimate the annual incidence of encephalitis in Ontario by cause category for 2002–2013, compare incidence rates between Ontario and England, and identify whether an association exists between encephalitis cause category and length of hospitalization. Public Health Ontario (Ontario Agency for Health Protection and Promotion) Research Review Board provided ethics approval for this study.

## Methods

### Data Source

We extracted hospital discharge diagnoses data from the Canadian Institute for Health Information (http://www.cihi.ca), Ontario Discharge Abstract Database, for April 2002–December 2013 through Ontario Ministry of Health and Long-Term Care’s IntelliHEALTH Ontario. The Ontario Discharge Abstract Database used ICD-10 during this period. We obtained ICD-10 codes for encephalitis diagnoses by reviewing similar studies (*4*,*15*,*16*). An encephalitis-associated hospitalization was defined as a hospitalization for which an encephalitis diagnostic code or specified combination of encephalitis codes were recorded in any of the diagnostic fields, including the field for the most responsible diagnosis (most responsible for the length of hospitalization), as done elsewhere (*15*).

We categorized ICD-10 codes into 8 categories of encephalitis cause: viral, bacterial, amebic, fungal, immune-mediated, parasitic, other, and unknown. We used a ninth category for cases that could not be categorized because of contradictory encephalitis-related ICD codes attributed to a single case. Multiple encephalitis hospitalizations for the same patient that occurred within 6 months (e.g., <6 months between the first discharge and second admission with an encephalitis ICD code in any diagnostic field) were considered 1 admission (*15*,*17*). In this situation, lengths of stay for the 2 hospitalizations were totaled into a single length of stay for the encephalitis patient. If the time between the first discharge and second admission was >6 months, the hospitalizations were considered unique visits and unique cases of encephalitis. Thus, we counted incident encephalitis-associated hospitalizations for a given patient with multiple admissions when the hospitalizations occurred >6 months apart. ICD-10 codes for immunosuppression were identified through a review of other studies and were related to having HIV, organ transplantation, immunodeficiency, or cancer (*7*,*18*).

### Data Extraction

We selected ICD-10 codes using the first 3 characters (e.g., B00) in any diagnostic field corresponding to encephalitis conditions. Filters were then implemented to extract specific 4-character (e.g., B004) encephalitis ICD-10 codes, both single codes and code combinations, that were recorded upon diagnosis of an encephalitis case (Technical Appendix).

### Analysis

Data were analyzed by using SAS version 9.3 (SAS Institute Inc., Cary, NC, USA). Incident cases of encephalitis were stratified by year of patient hospital admission; sex; age at admission (<1, 1–4, 5–19, 20–44, 45–64, >65 years of age); and geography (patient Local Health Integration Network [LHIN]). Hospitalization rates for incident all-cause encephalitis were calculated overall, by year and patient sex, age group, and LHIN by using yearly Ontario population estimates from Statistics Canada (http://www.statscan.ca) CANSIM tables. We calculated 95% CIs for incidence densities through bootstrap resampling with 4,000 repetitions. We also calculated incidence rates and 95% CIs by category of encephalitis cause, stratified by year, sex, and age. These values were compared with incidence rates from studies conducted in England (*15*). We calculated proportions and frequency counts of discharges by specific encephalitis cause for their respective cause categories and for encephalitis in Ontario as a whole.

After applying incidence estimates for England from April 1, 2005, through March 31, 2009 (2005–2008 fiscal years), to Ontario population data, we determined the expected case counts for each sex and age group if the age/sex incidence of encephalitis in Ontario was the same as in England. We compared these expected case counts on the basis of incidence rate data in England with the actual case counts of encephalitis in Ontario during these fiscal years.

Yearly and seasonal trends in hospital discharges from incident all-cause encephalitis were investigated by regression analyses adjusted for age and sex. The outcome variable was the number of incident encephalitis-associated hospitalizations in Ontario. We applied negative binomial regression with an overdispersion parameter that captured the heterogeneity among observations that could not be accounted with Poisson model. The logarithm of the population at risk, the Ontario population, was included as an offset in this model. Single predictor and multivariable negative binomial regression models were performed; the latter was adjusted for age, sex, and year.

We used multiple linear regression to assess the association between length of hospital stay for a patient with an encephalitis-associated admission (continuous variable) and encephalitis cause (a 7-category variable for type of encephalitis cause: viral, bacterial, immune-mediated, amebic/parasitic/fungal, other, unknown, and unable to classify). The length of hospitalization outcome variable was natural log transformed to ensure it was normally distributed in this linear regression model. To enable the log transformation, we recorded all hospitalizations of <1 day (0 days) as 0.5 days because of a lack of precise information about admission and discharge times. Using descriptive analysis, we explored the mean and median length of hospitalization for the different groups of encephalitis cause. Unadjusted associations and associations adjusted for sex and age were calculated. We then adjusted for the baseline model that included age and sex by clinically relevant predictors of the outcome and confounders of the association.

## Results

### Incidence

During April 2002–December 2013, incidence of all-cause encephalitis was ≈4.3 (95% CI 4.2–4.4) cases/100,000 persons per year in Ontario. Encephalitis occurred more frequently among male than female Ontario residents in all age groups except children 1–4 years of age (Table 1). The youngest and oldest age groups had the highest incidence of encephalitis; for infants <1 year of age, incidence was 10.7 (95% CI 9.1–12.1) cases/100,000 population, and for persons >65 years of age, incidence was 8.1 (95% CI 7.9–8.6) cases/100,000 population. These trends were consistent during the entire 12-year study period; encephalitis peaked in infants in 2004 (18.7 [95% CI 12.0–26.2] cases/100,000 persons) and in elderly persons in 2002 (14.1 [95% CI 12.1–16.4] cases/100,000).

**Table 1:** Encephalitis incidence rates, Ontario, Canada, 2002–2013

**Year**	**Total population (95% CI)**	Incidence (95% CI)*								
		Sex			Age group, y					
		M	F		<1	1–4	5–19	20–44	45–64	≥65
**2002**	6.0 (5.6–6.6)	6.4 (5.6–7.2)	5.7 (5.0–6.4)		10.3 (4.1–17.6)	6.1 ( 4.0–8.5)	4.7 (3.8–5.7)	3.6 (3.0–4.3)	6.6 (5.5–7.7)	14.1 (12.1–16.4)
**2003**	3.9 (3.6–4.3)	4.2 (3.6–4.7)	3.8 (3.3–4.2)		6.9 (3.1–12.3)	6.3 (4.3–8.5)	3.6 (2.8–4.4)	2.3 (1.9–2.8)	4.3 (3.5–5.0)	7.6 (6.2–9.1)
**2004**	3.7 (3.3–4.0)	3.5 (3.0–4.0)	3.9 (3.4–4.4)		18.7 (12.0–26.2)	4.6 (2.9–6.4)	2.9 (2.3–3.6)	2.6 (2.1–3.0)	4.0 (3.4–4.8)	5.7 (4.6–6.9)
**2005**	4.0 (3.6–4.3)	4.2 (3.7–4.7)	3.8 (3.3–4.3)		9.0 (4.5–14.2)	5.3 (3.5–7.3)	2.7 (2.1–3.4)	2.5 (2.1–3.0)	4.5 (3.8–5.3)	8.0 (6.6–9.4)
**2006**	3.8 (3.5–4.2)	4.3 (3.8–4.8)	3.3 (2.9–3.8)		10.2 (5.1–16.0)	3.8 (2.2–5.4)	2.9 (2.2–3.6)	2.6 (2.1–3.7)	3.9 (3.2–4.6)	7.9 (6.7–9.3)
**2007**	3.9 (3.5–4.2)	4.4 (3.9–4.9)	3.4 (3.0–3.9)		11.0 (5.9–16.8)	5.9 (3.9–8.1)	2.7 (2.1–3.4)	2.6 (2.2–3.1)	4.3 (3.6–5.0)	7.1 (5.8–8.3)
**2008**	3.9 (3.6–4.3)	4.6 (4.1–5.1)	3.3 (2.8–3.2)		12.1 (7.1–18.2)	4.5 (2.9–6.2)	2.8 (2.2–3.5)	2.8 (2.4–3.3)	4.2 (3.5–4.9)	6.9 (5.6–8.1)
**2009**	4.0 (3.7–4.3)	4.4 (3.9–4.9)	3.6 (3.2–4.1)		10.0 (5.0–15.7)	6.5 (4.4–8.8)	2.7 (2.1–3.4)	2.8 (2.3–3.2)	3.8 (3.2–4.4)	7.8 (6.5–9.1)
**2010**	4.0 (3.6–4.3)	4.1 (3.6–4.6)	3.8 (3.4–4.3)		5.7 (2.1–10.0)	6.5 (4.4–8.7)	3.2 (2.5–3.9)	2.8 (2.3–3.3)	4.1 (3.5–4.7)	6.8 (5.6–8.0)
**2011**	4.7 (4.3–5.0)	5.0 (4.5–5.6)	4.3 (3.8–4.8)		8.6 (4.3–13.6)	6.1 (4.2–8.1)	3.1 (2.4–3.8)	2.9 (2.4–3.4)	5.5 (4.7–6.2)	8.5 (7.2–9.9)
**2012**	5.4 (5.0–5.8)	5.8 (5.2–6.4)	4.9 (4.4–5.5)		12.1 (6.4–18.5)	6.1 (4.2–8.1)	4.4 (3.6–5.2)	3.3 (2.8–3.8)	5.4 (4.8–6.2)	10.5 (9.1–12.0)
**2013**	4.6 (4.2–5.0)	4.8 (4.3–5.3)	4.4 (3.8–4.9)		14.1 (8.5–20.4)	6.8 (4.7–8.9)	3.4 (2.7–4.3)	2.7 (2.2–3.1)	4.4 (3.8–5.1)	9.3 (8.0–10.6)
**Overall**	4.3 (4.2–4.4)	4.6 (4.4–4.8)	4.0 (3.8–4.1)		10.7 (9.1–12.1)	5.7 (5.2–6.3)	3.2 (3.0–3.4)	2.8 (2.6–2.9)	4.4 (4.3–4.7)	8.3 (7.9–8.6)

*Incidence is number of cases/100,000 persons. 95% Cis were calculated through bootstrap resampling encephalitis case counts for each age, sex, and year by using 4,000 repetitions.

The incidence of all-cause encephalitis peaked for both male and female residents in August and September 2002 (96 and 140 cases/100,000 persons, respectively) and 2012 (101 and 85 cases/100,000 persons, respectively). Otherwise, we observed no linear time trend during the 12-year study period (p = 0.9). In general, during July–October, incidence rates were higher by age group for infants and for persons >65 years of age; for other age groups, encephalitis incidence remained relatively constant throughout the year.

The incidence of immune-mediated encephalitis was highest in children 1–4 years of age (0.7 cases/100,000 persons) (Figure). The incidence of viral encephalitis and encephalitis of unknown cause was highest in infants <1 year of age, followed by adults >65 years of age.

**Figure:**
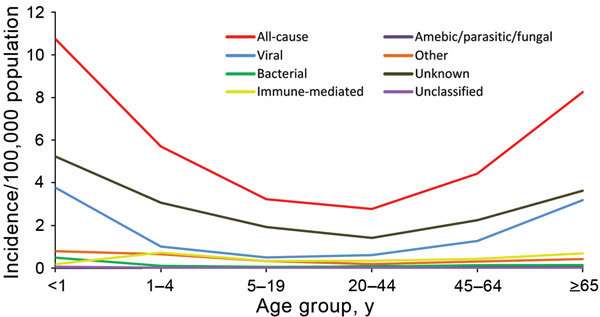
Incidence rate (cases per 100,000 persons) for all-cause encephalitis and categories of encephalitis causes, by age group, Ontario, Canada, 2002–2013.

### Immunocompetent and Immunocompromised Persons with Encephalitis

The 938 immunocompromised patients with encephalitis received the following ICD-10 codes at hospital discharge: 65.4%, a code indicating cancer; 27.9%, a code indicating HIV infection; 12.4%, a code indicating transplantation; and 3.4%, a code indicating immunodeficiency (Table 2). Fifty-one percent of encephalitis patients with HIV, 40.6% with immunodeficiency, 44.8% who had undergone transplantation, and 28.1% with cancer had viral encephalitis. Sixty (22.9%) of encephalitis cases among persons with HIV were amebic/parasitic/fungal encephalitis, which was more than twice the proportion of these causes among other immunocompromised persons. Among encephalitis patients with cancer, 32.1% had immune-mediated encephalitis; for 28.2%, encephalitis cause was unknown. Among immunocompromised persons with HIV, immunodeficiency, or a transplantation, the most common encephalitis cause, other than viral, was unknown cause.

**Table 2:** Cause of encephalitis in immunocompromised patients, Ontario, Canada, 2002–2013

**Encephalitis cause**	**Total encephalitis cases, no. (%), N = 6,463**	**Immunocompromising condition, no. (%), n = 938**			
		HIV, n = 262	Other immunodeficiency, n = 32	Transplant, n = 116	Cancer, n = 613
**Unknown**	3,299 (51.0)	45 (17.2)	9 (28.1)	42 (36.2)	176 (28.7)
**Viral**	1,788 (27.7)	134 (51.2)	13 (40.6)	52 (44.8)	172 (28.1)
**Immune mediated**	657 (10.2)	3 (1.2)	5 (15.6)	7 (6.0)	197 (32.1)
**Other**	466 (7.2)	11 (4.2)	2 (6.3)	5 (4.3)	42 (6.9)
**Bacterial**	152 (2.4)	7 (2.7)	0	2 (1.7)	13 (2.1)
**Amebic/parasitic/fungal**	92 (1.4)	60 (22.9)	3 (9.4)	8 (6.9)	12 (2.0)
**Unable to classify**	9 (0.1)	2 (0.8)	0	0	1 (0.2)
**Total**	6,463	262 (27.9)	32 (3.4)	116 (12.4)	613 (65.4)

Encephalitis cause was unknown for 55.2% of immunocompetent patients and for 26.6% of immunocompromised patients. A total of 35.6% of immunocompromised persons and 26.3% of immunocompetent persons had viral encephalitis, a difference of 9.3%. For immune-mediated encephalitis, the difference was 13.6% (21.8% for immunocompromised vs. 8.2% for immunocompetent patients); for amebic/parasitic/fungal causes, the difference was 7.1% (7.5% for immunocompromised vs. 0.4% for immunocompetent patients).

The mean log-transformed length of hospitalization for encephalitis, as determined by discharge data, was significantly longer for immunocompromised than immunocompetent patients (p<0.0001). The 32 persons in whom immunodeficiency was diagnosed had the widest range of hospitalization stay, and the 116 persons who had an organ transplant had the longest median hospitalization stay (22.5 days), of all subcategories of persons with immunocompromising conditions. For both immunocompromised and immunocompetent persons, bacterial encephalitis resulted in the longest hospital stays (34.5 and 16.5 days, respectively). Among encephalitis cases we were able to classify, encephalitis of unknown cause resulted in the shortest hospital stays for both immunocompromised (18 days) and immunocompetent (9 days) patients, even though stay was twice as long for immunocompromised patients.

Overall, during 2002–2013, age- and year-adjusted encephalitis incidence was 15% higher for male patients (4.6 [95% CI 4.4–4.8] cases/100,000 persons) than for female patients (4.0 [95% CI 3.8–4.1] cases/100,000 persons) (Table 3). Sex- and year-adjusted encephalitis incidence for infants was 3.9 (95% CI 3.3–4.5) times greater than for adults 20–44 years of age (considered the referent category because this group had the lowest incidence), and sex- and year-adjusted encephalitis incidence for adults >65 years of age was 3.0 (95% CI 2.8–3.2) times that of adults 20–44 years of age (p<0.0001). Incidence rate ratios of Ontario and England by age and sex did not appear to differ substantially, except for the oldest age group. In multivariable models, compared with adults in the 20–44-year age category, persons >65 years of age in Ontario had an incidence rate ratio of 3.0 (95% CI 2.8–3.2) versus a significantly lower incidence rate ratio of 1.9 (95% CI 1.8–2.1) for this age group in England.

**Table 3:** Univariable and multivariable negative binomial regression model assessing variation in incident encephalitis hospitalizations, Ontario, Canada, and England*

**Variable**	**No. (%) cases, N = 6,463**	**Incidence rate**	**Multivariable analysis**				
			Ontario, 2002–2013			England, 2005–2009†	
			Adjusted IRR (95% CI)	p value		Adjusted IRR (95% CI)	p value
**Sex**							
M	3,417 (52.8)	4.6	Referent	<0.0001		Referent	0.002
F	3,046 (47.1)	4.0	0.9 (0.8–0.9)			0.9 (0.9–1.0)	
**Age group, y**							
<1	173 (2.7)	10.7	3.9 (3.3–4.5)	<0.0001		3.7 (3.2–4.2)	<0.001
1–4	377 (5.8)	5.7	2.1 (1.8–2.3)			1.9 (1.7–2.1)	
5–19	915 (14.2)	3.2	1.2 (1.1–1.3)			0.9 (0.8–1.0)	
20–44	1,486 (23.0)	2.8	Referent			Referent	
45–64	1,823 (28.2)	4.4	1.6 (1.5–1.8)			1.4 (1.3–1.5)	
**>65**	1,689 (26.1)	8.3	3.0 (2.8–3.2)			1.9 ( 1.8–2.1)	

*Incidence is number of cases/100,000 persons. IRR, incident rate ratio. **†Reference (*****1*****).**

### Comparison of Encephalitis Cases in Ontario and England

In Ontario, the annual total number of encephalitis cases fell within the 95% CIs for the England-derived Ontario expected case counts in the 2005, 2007, and 2008 fiscal years. During the 2006 fiscal year, the number of cases in Ontario was lower than the estimated number expected on the basis of incidence rates in England. Overall, during April 2005–March 2009, the actual average per year case count of encephalitis in Ontario was 494 cases, which is not significantly different from the number of cases that would occur if England incidence rates were applied to the Ontario population (550 [95% CI 476–631] cases). During this period, encephalitis occurred significantly less often in female patients in Ontario (220 cases) than in England (268 [95% CI 233–307] cases). For adults >65 years of age, encephalitis occurred significantly more often in Ontario (126 cases) than in England (102 [95% CI 89–113] cases). In England, the proportion of encephalitis cases in immunocompromised patients as identified by a population-based prospective study was 15.3%, and in Ontario, 14.5% (*7*).

### Encephalitis Cause and Length of Hospitalization

The multiple linear regression model exploring the association between category of encephalitis cause and length of hospitalization was adjusted by sex, age, immune status, and co-morbidity level, all of which resulted in a >20% change in the parameter coefficients from the baseline model (Table 4). Season, year, and patient LHIN did not significantly change (>20%) in the parameter estimates for the baseline model (which included age and sex in addition to main exposure and outcome) and were thus excluded from the model. After adjusting for all significant covariates of interest, we found that patients with amebic/parasitic/fungal encephalitis had a 27.5% (95% CI 1.4%–60.4%) longer hospital stay than did patients with viral encephalitis (p = 0.038). In addition, after adjusting for all covariates of interest, we found length of hospitalization to be 22.1% (95% CI 17.0%–26.8%) shorter for patients with encephalitis of unknown cause than for patients with viral encephalitis.

**Table 4:** Multiple linear regression modeling association between log-transformed length of hospitalization and category of encephalitis cause, Ontario, Canada, 2002–2013

Variable*	**No. (%) cases**	**Mean length of hospitalization, d (median)**	**Exponentiated β-coefficient (95% CI)**	**t**	**p value**
**Intercept**			8.9 (8.0–9.8)	41.0	<0.0001
**Encephalitis cause**					
Viral	1,788 (27.7)	27.36 (14)			
Bacterial	152 (2.4)	27.45 (19)	1.2 (1.0–1.4)	1.5	0.128
Amebic/parasitic/fungal	92 (1.4)	43.17 (18)	1.3 (1.0–1.6)	2.1	0.038
Immune mediated	657 (10.2)	26.37 (14)	1.1 (1.0–1.2)	1.8	0.0804
Other	466 (7.2)	24.33 (11)	0.9 (0.8–1.0)	−1.7	0.0817
Unknown	3,299 (51.0)	19.79 (9)	0.8 (0.7–0.8)	−7.8	<0.0001
Unable to classify	9 (0.1)	38.78 (16)	2.1 (1.0–4.1)	2.0	0.0448
**Sex**					
M	3,417 (52.8)	23.07 (11)			
F	3,046 (47.1)	23.82 (12)	1.0 (1.0–1.1)	0.9	0.3634
**Age group, y**					
<1	173 (2.7)	22.83 (16)	1.0 (0.8–1.1)	−0.6	0.55
1–4	377 (5.8)	12.28 (5)	0.6 (0.5–0.7)	−8.6	<0.0001
5–19	915 (14.2)	14.54 (6)	0.8 (0.8–0.9)	−8.4	<0.0001
20–44	1,486 (23.0)	23.27 (9)	0.8 (0.8–0.9)	−3.6	0.0003
45–64	1,823 (28.2)	25.02 (13)			
**>65**	1,689 (26.1)	29.19 (17)	1.1 (1.1–1.2)	3.7	0.0003
**Immune status**					
Immunocompetent	5,525 (85.5)	21.29 (10)			
Immunocompromised	938 (14.5)	35.99 (19)	1.3 (1.2–1.4)	5.6	<0.0001
**Co-morbidity level†**					
None	3718 (57.5)	14.05 (8)			
Low	564 (8.7)	26.08 (14)	1.7 (1.6–1.9)	11.0	<0.0001
Moderate	911 (14.1)	26.13 (15)	1.8 (1.7–2.0)	14.8	<0.0001
High	575 (8.9)	39.00 (25)	2.8 (2.6–3.1)	21.2	<0.0001
Very high	520 (8.1)	55.28 (36)	4.0 (3.6–4.4)	27.0	<0.0001
Missing data	175 (2.7)	14.05 (41)	4.4 (3.7–5.2)	17.9	<0.0001

*Season, year, and patient Local Health Integration Network were not found to cause a significant change (>20%) in the parameter estimates for the exposure variable (category of encephalitis cause) and were thus excluded from the mode. **†Case mix grouping plus comorbidity levels are based on cumulative cost impact of comorbidities on patient stay, where "none" represents no impact and "very high” represents the greatest impact.**

Length of hospitalization did not differ significantly by patient sex (p = 0.3634) but was 25.3% longer for immunocompromised than for immunocompetent patients (p<0.0001). After adjustment, compared with results for adults 45–64 years of age, average hospitalization was 40.8% (95% CI 33.2%–47.5%) shorter for children 1–4 years of age, 16.9% (95% CI 12.2%–20.4%) shorter for children and youth 5–19 years of age, 12.6% (95% CI 5.9%–18.8%) shorter for adults 20–44 years of age, and 14.2% (95% CI 6.4%–22.7%) longer for adults >65 years of age. All levels of co-morbidity were associated with significantly longer hospitalization (p<0.0001) than was lack of any co-morbidities.

## Discussion

Our findings regarding the epidemiology of encephalitis in Ontario are similar to those identified in previous studies in Canada, the United States, and England and update the incidence of encephalitis in Ontario and its causal distribution (*3*,*4*,*17*,*19*). In particular, we found results similar to those from England, in relation both to the proportion of encephalitis cases of unknown cause and incidence by patient age and sex, despite the occurrence of zoonotic viral infections in Ontario that are not found in England. These findings imply that most infectious causes are likely to be globally distributed with similar epidemiology in both England and Ontario, not clustering in particular locations or in large outbreaks. Alternatively, a similarly broadly distributed noninfectious cause might be responsible, such as an immune-mediated cause that has been more recently discovered or that is yet unidentified. The shorter hospital stay for persons with encephalitis of unknown cause also might indicate that some cases are not actually encephalitis. This information will provide baselines for future studies, as new diagnostic methods become available, examining changes in the distribution of encephalitis cases by cause and studies evaluating trends in encephalitis incidence over time.

Limitations exist to the use of administrative data to describe epidemiology. We were unable to validate the diagnoses and did not have access to additional laboratory testing information or specimens, which prevented us from identifying and correcting any possible coding errors (*9*). In England, this limitation was addressed through a study of encephalitis, one of the largest population-based studies that exists (*20*). We also were unable to control the diagnostic testing methods used by physicians in Ontario and could only assume that physicians followed provincial standards to derive encephalitis diagnoses. Because of the use of administrative data, misclassification bias also is highly possible, particularly because specific causes of encephalitis often are difficult to diagnose, and whether cases identified are truly incident cases and not sequelae remaining long after infection is unclear. Because we used all diagnostic fields, not solely the primary diagnostic field, to identify encephalitis cases, we could be overestimating the number of cases in persons admitted for sequelae. In some cases, assigning a diagnostic code from information available in the administrative dataset is difficult. We found 329 encephalitis patients who had multiple hospitalizations <6 months apart that did not have the same ultimate encephalitis diagnosis decision for each hospitalization. Of these cases, 320 had multiple encephalitis diagnoses from different hospitalizations that were in the same cause category as previously defined. The remaining 9 cases were categorized as “unable to classify.” Last, our study included cases for which encephalitis was listed as the most responsible diagnosis and cases for which it was listed as a secondary reason for hospital admission. We were unable to test whether this measure confounded the association between encephalitis cause and length of hospitalization.

Several possible reasons explain why there are encephalitis patients with multiple hospitalizations that have different encephalitis cause diagnoses. First, we analyzed administrative data that might have ICD-10 coding errors, resulting in conflicting encephalitis diagnosis decisions for the same patient within a 6-month period. Second, given the difficult task of diagnosing encephalitis, and more specifically identifying the specific type of encephalitis, for patients rehospitalized for encephalitis within a 6-month period it is possible that the initial diagnosis was incorrect, and that the subsequent diagnosis was more accurate.

This study has several strengths. The study was not conducted solely during an outbreak, so it is not biased toward a particular cause. Data were collected and analyzed from the entire province, and geography was tested as an important confounder of the main association by the proxy variable of the LHIN in which the patient resides. Use of discharge data also prevented double counting of patients who were transferred between hospitals, an important and common occurrence for encephalitis patients who might need tertiary care facilities.

The results from this study increases understanding of encephalitis incidence in Ontario. These results can be used as a baseline for future studies to identify changes in encephalitis over time and changes in the distribution of causes of encephalitis to identify emerging diseases that are initially likely to be categorized as being of unknown cause. These findings also suggest that under-ascertainment of encephalitis cases is similar in Ontario and England or does not occur. Better understanding the association between encephalitis cause and length of hospitalization can help target interventions, and these data can be used to help advocate for increased use of personal protective devices against mosquitoes and ticks, which are major vectors of encephalitis in Ontario. An understanding of the epidemiology of encephalitis in Ontario is beneficial in public health surveillance of emerging infectious diseases. Similarities between the epidemiology of encephalitis in Ontario and England, despite differences such as the presence of West Nile virus in Ontario, imply that infectious causes of encephalitis are most likely to be widespread and non-epidemic pathogens, or alternatively, not infectious diseases at all.

Technical Appendix International Classification of Diseases, Tenth Revision diagnostic codes for identifying encephalitis and etiologic groupings; data extraction process flowchart for patients hospitalized with encephalitis, Ontario, Canada.
