# Supplementary material for: Encephalitis, Ontario, Canada, 2002–2013
Source: Emerg Infect Dis. 2016 Mar;22(3):426–32. doi: 10.3201/eid2203.151545 (PMC4766895; doi:10.3201/eid2203.151545)

Article DOI: http://dx.doi.org/10.32032/eid2203.151545

Encephalitis, Ontario, Canada, 2002–2013

Technical Appendix

Technical Appendix Table. International Classification of Diseases, Tenth Revision (ICD-10) diagnostic codes for identifying encephalitis and etiologic groupings

Encephalitis Diagnosis and Etiology Category Groupings		ICD-10 Code
Viral		
Herpesviral encephalitis: Herpesviral meningoencephalitis, Simian B disease		B004*
Varicella Zoster Virus = B011* OR B020*	Varicella encephalitis: Postchickenpox encephalitis, Varicella encephalomyelitis	B011*
	Zoster encephalitis: Zoster meningoencephalitis	B020*
Enteroviral encephalitis: Enteroviral encephalomyelitis		A850*
Adenoviral encephalitis: Adenoviral meningoencephalitis		A851*
Measles complicated by encephalitis		B050*
Mumps encephalitis		B262*
Rabies = A820* OR A821* OR A829*	Sylvatic Rabies	A820*
	Urban Rabies	A821*
	Rabies, unspecified	A829*
Arboroviruses = A830* OR A831* OR A832* OR A833* OR A834* OR A835* OR A836* OR A922* OR A838* OR A839* OR A852* OR A840* OR A841* OR A848* OR A849*	Japanese encephalitis	A830*
	Western equine encephalitis	A831*
	Eastern equine encephalitis	A832*
	St Louis encephalitis	A833*
	Australian encephalitis	A834*
	California encephalitis	A835*
	Rocio virus disease	A836*
	Other mosquito-borne viral encephalitis	A838*
	Mosquito-borne viral encephalitis, unspecified	A839*
	Venezuelan equine fever	A922*
	Arthropod-borne viral encephalitis, unspecified	A852*
	Far Eastern tick-borne encephalitis [Russian spring-summer encephalitis]	A840*
	Central European tick-borne encephalitis	A841*
	Other tick-borne viral encephalitis	A848*
	Tick-borne viral encephalitis, unspecified	A849*
Rubella with viral encephalitis		B060* AND G051*
Influenza with viral encephalitis		(J108* OR J118*) AND G051*
Polio: Acute paralytic poliomyelitis, vaccine-associated or Acute paralytic poliomyelitis, wild virus, imported or Acute paralytic poliomyelitis, wild virus, indigenous or Acute paralytic poliomyelitis, other and unspecified or Acute nonparalytic poliomyelitis or Acute poliomyelitis, unspecified; with viral encephalitis		(A800* OR A801* OR A802* OR A803* OR A804* OR A809*) AND G051*
Lymphocytic meningoencephalitis		A872* AND G051*
Cytomegaloviral encephalitis		B258* AND G051*
HIV disease resulting in encephalitis		B220* AND G051*
Progressive multifocal leukoencephalopathy		A812*
Other specified viral encephalitis: Encephalitis lethargica; Von Economo-Cruchet disease		A858*
Subacute sclerosing panencephalitis: Dawson inclusion body encephalitis; Van Bogaert sclerosing leukoencephalopathy		A811*
West Nile virus infection: West Nile fever		A923*
Bacterial		
Listerial meningoencephalitis		A321* AND G050*
Meningococcal encephalitis		A398* AND G050*
Late congenital syphilitic encephalitis and late syphilitic encephalitis		(A504* OR A521*) AND G050*
Tuberculosis meningoencephalitis		A178* AND G050*
Streptococcal encephalitis		A491* AND G050*
Lyme disease encephalitis: Erythema chronicum migrans due to Borrelia burgdorferi encephalitis		A692* AND G050*
Cat-scratch encephalitis: cat scratch encephalitis fever		A281* AND G050*
Actinomycosis encephalitis		A428* AND G050*
Bacterial meningoencephalitis and meningomyelitis, not elsewhere classified		G042*
Parasitic		
Trypanosomiasis = B560*OR B561*OR	Gambiense trypanosomiasis: Infection due to Trypanosoma brucei gambiense; West African sleeping sickness	B560*

Encephalitis Diagnosis and Etiology Category Groupings		ICD-10 Code
Viral		
B569*OR B574*	Rhodesiense trypanosomiasis: East African sleeping sickness; Infection due to Trypanosoma brucei rhodesiense	B561*
	African trypanosomiasis, unspecified: Sleeping sickness NOS; Trypanosomiasis NOS in places where African trypanosomiasis is prevalent	B569*
Chagas disease (chronic) with nervous system		B574*
Toxoplasma meningoencephalitis		B582*
Amoebic		
Naegleriasis: Primary amoebic meningoencephalitis		B602*
Angiostrongyliasis due to Parastrongylus cantonesis: Angiostrongyliasis due to Angiostrongylus cantonensis; Eosinophilic meningoencephalitis		B832*
Fungal		
Cerebral cryptococcosis encephalitis		B451* AND G052*
Immune-Mediated		
Acute disseminated encephalitis: postimmunization encephalitis and encephalomyelitis		G040*
Acute and subacute haemorrhagic leukoencephalitis [Hurst]		G361*
Systemic lupus erythematosus encephalitis with organ or system involvement		M321* AND G058*
Paraneoplastic limbic encephalopathy		G131*
Mixed Other		
Mixed other groups: other encephalitis, myelitis, and encephalomyelitis; postinfectious encephalitis and encephalomyelitis not otherwise specified.		G048*
Unknown		
Unspecified encephalitis, myelitis, and encephalomyelitis (viral, bacterial, infectious, parasitic)		A86* OR G050*OR G051*OR G052*OR G058* IF no other code specified
Encephalitis, myelitis and encephalomyelitis, unspecified: Ventriculitis (cerebral) NOS		G049*

Technical Appendix Figure. Data extraction process flowchart for patients hospitalized with encephalitis, Ontario, Canada. ICD-10, International Classification of Diseases, Tenth Revision.

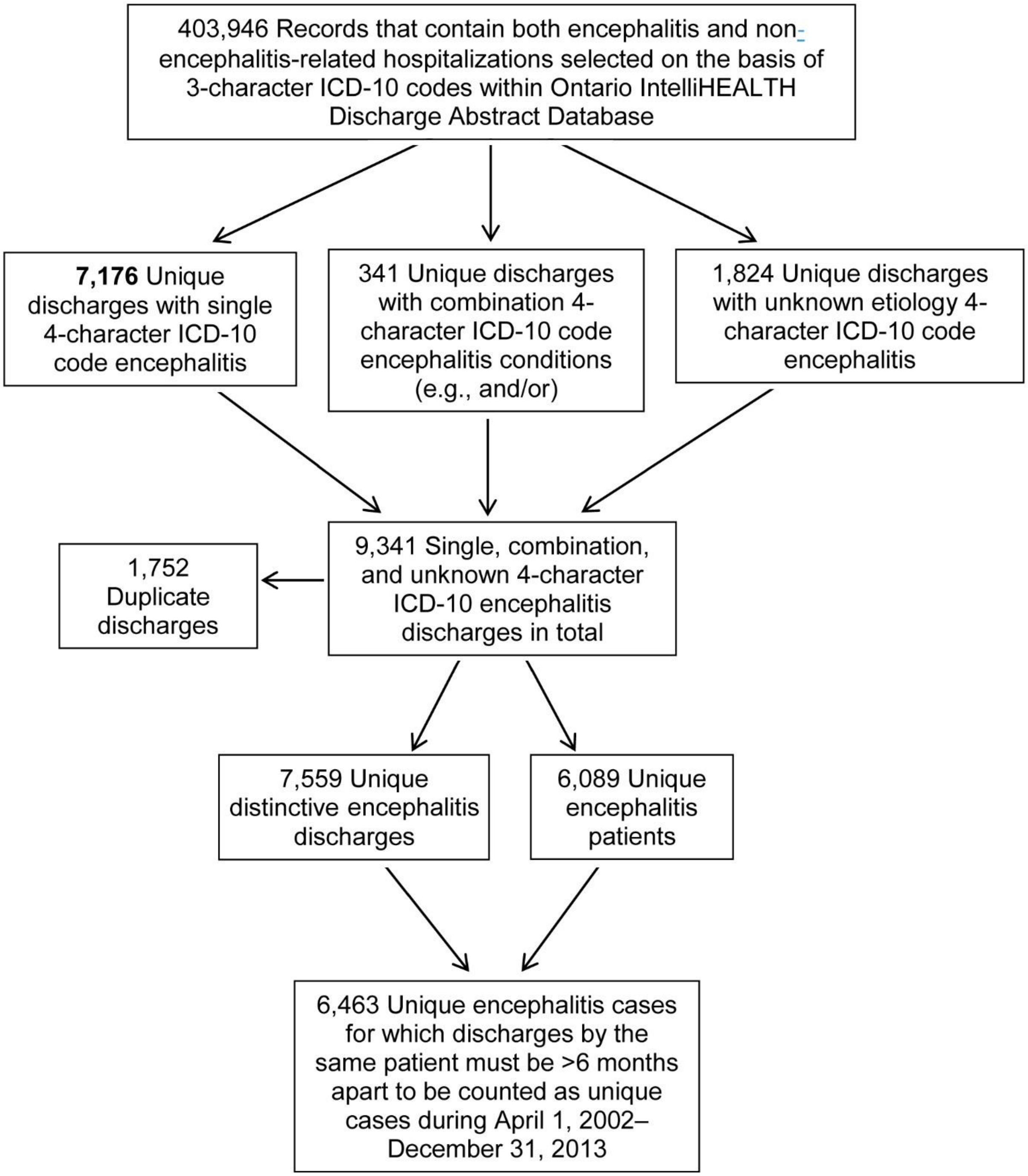

Supplement: Technical Appendix — International Classification of Diseases, Tenth Revision diagnostic codes for identifying encephalitis and etiologic groupings; data extraction process flowchart for patients hospitalized with encephalitis, Ontario, Canada. [file 15-1545-Techapp-s1.pdf]
